# Blood pressure, antihypertensive medication and neuropsychiatric symptoms in older people with dementia: The COSMOS study

**DOI:** 10.1002/gps.5388

**Published:** 2020-10-08

**Authors:** Bianca E. M. de Jong‐Schmit, Rosalinde K. E. Poortvliet, Stefan Böhringer, Jonathan M. K. Bogaerts, Wilco P. Achterberg, Bettina S. Husebo

**Affiliations:** ^1^ Department of Public Health and Primary Care Leiden University Medical Center Leiden The Netherlands; ^2^ Department of Medical Statistics and Bioinformatics Leiden University Medical Center Leiden The Netherlands; ^3^ Department of Global Public Health and Primary Care Centre for Elderly and Nursing Home Medicine, University of Bergen Bergen Norway

**Keywords:** antihypertensive medication, blood pressure, dementia, neuropsychiatric symptoms

## Abstract

**Objectives:**

Neuropsychiatric symptoms (NPS) are very common in older patients with dementia. There is increasing evidence that hypoperfusion of the brain plays a role in the development of NPS. The aim of this study is to assess whether there is an association between low systolic blood pressure (SBP) and NPS and if NPS are more prevalent in older people with dementia using antihypertensive medication.

**Methods:**

We studied the baseline data from participants in the *Co*mmunication, *S*ystematic pain treatment, *M*edication review, *O*rganized activities and *S*afety study, a multicenter clustered trial with 765 participants from 72 nursing home units from 37 nursing homes in Norway. SBP (lowest quartile vs rest) and use of antihypertensive medication were predictors and Neuropsychiatric Inventory—Nursing Home version (NPI‐NH) score (total and clusters) was the outcome. Missing data were imputed, except for missing data in predictors. We used a mixed model analysis adjusted for age, sex and Minimal Mental State Examination (MMSE) score. In a sensitivity analysis, continuous SBP values were used.

**Results:**

In total, 412 patients were included with a mean age of 86.9 years, 53.9% had a MMSE score of <11. There was no difference in total NPI‐NH score between low and high SBP (difference −1.07, *P*_dj_ = 0.62). There was no difference between high and low SBP and the NPI clusters. The use of antihypertensive medication was not associated with a different total or cluster NPI‐NH score compared to no use (difference −0.99, *P*_adj_ = 0.95, *P*_all_ = 0.37‐0.99, respectively). In the sensitivity analyses with the continuous SBP levels, there was no association between SBP and NPI‐NH score (estimate 1.00, 95%CI 0.98‐1.01, *P* = 0.25).

**Conclusion:**

We found no association between low SBP and NPS, nor between antihypertensive use and NPS.

**Key points**

- Neuropsychiatric symptoms are very common in older patients with dementia and clinical management is challenging. The etiology of NPS is multifactorial.
- There is increasing evidence that hypoperfusion of the brain plays a role in the development of neuropsychiatric symptoms.
- No evidence was found between a low systolic blood pressure and neuropsychiatric symptoms in this nursing home population.
- On the contrary to earlier studies, we found no association between neuropsychiatric symptoms and the use of antihypertensive medication.

## 1 INTRODUCTION

Neuropsychiatric symptoms (NPS), such as apathy, delusions, hallucinations, agitation and aggressive behavior, are very common in older people with dementia.^1^, ^2^ About 90% of the people with dementia experience at least one of these symptoms over the course of their illness.^3^, ^4^ With advanced stages of dementia in time, the severity of NPS will change with especially a more severe degree of agitation, depression, apathy and anxiety.^5^, ^6^ NPS are highly distressing for people with dementia and stressful for their caregivers.^3^, ^5^ In addition, NPS are also known to be associated with a poorer quality of life and increase the cost of care.^7^, ^8^ The clinical management of NPS is therefore a high priority for clinicians treating people with dementia,^3^ but the efficacy and safety of the available drug treatments are controversial.^3^, ^4^, ^8^, ^9^

The etiology of NPS in dementia is multifactorial and includes neuropathological changes in the brain as well as unmet physical and psychological needs related to dementia.^9^ One of the possible neuropathophysiological mechanisms is the observed relationship between hypoperfusion of the brain and NPS. There is increasing evidence that hypoperfusion of the brain plays a role in the development of NPS like apathy,^10^, ^11^, ^12^, ^13^, ^14^ depressive symptoms,^15^ psychotic symptoms^16^ and aggressive behavior.^17^ Hypertension and cardiovascular disease, such as stroke, can cause disturbances in the cerebrovascular hemodynamics and hypoperfusion of specific brain areas can occur somewhere in the course of their life.^18^ This suggests that disturbances in cerebrovascular hemodynamics followed by hypoperfusion of the brain may have an influence on NPS. In people with Alzheimer's disease and vascular dementia, it is known that they have pronounced disturbances in their cerebrovascular hemodynamics.^19^ As a consequence, blood pressure reduction in older people with impaired hemodynamic response may lead to hypoperfusion of the brain, resulting in increased mental health problems.^20^ Almost half of the nursing home residents is affected with cardiovascular diseases^21^ such as hypertension and therefore use antihypertensive medication. In people with dementia, the use of antihypertensive medication itself has been associated with increased occurrence of NPS^22^ and a greater progression in cognitive decline.^20^ The underlying mechanism of the found associations is unclear, but it is suggested that low blood pressure may lead to hypoperfusion of certain brain areas, that it is related to the use of the antihypertensive medication itself, or to the cumulative damage of the cardiovascular disease at older age.^20^, ^21^, ^22^

Given the above, a low systolic blood pressure (SBP) in persons with dementia might have a negative effect on the NPS. Therefore, we aim to assess whether there is an association between low blood pressure and NPS in persons with dementia and if NPS are more prevalent in persons with dementia using antihypertensive medication compared to persons with dementia not using antihypertensive medication.

## 2 MATERIALS AND METHODS

### 2.1 Design

**FIGURE 1:**
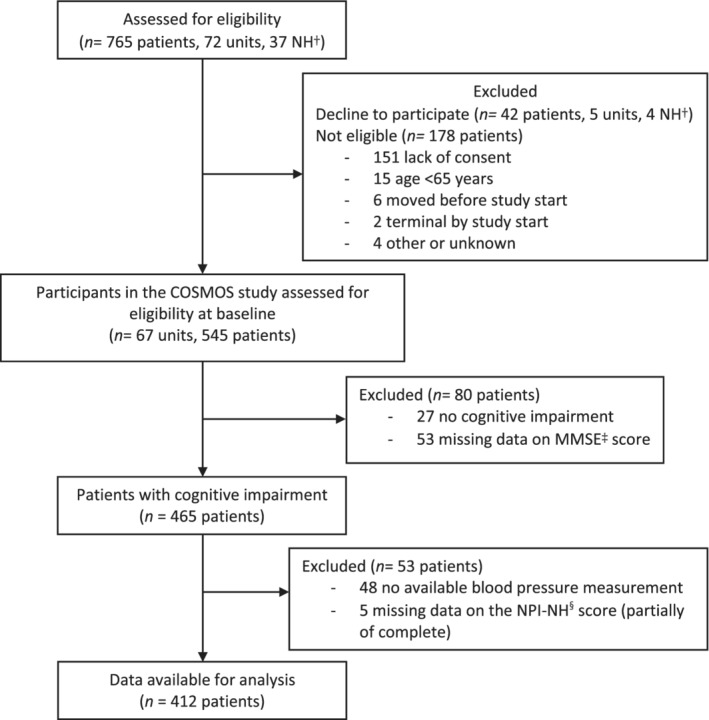
Flowchart of the study. †Nursing Home. ‡ MMSE = Minimal Mental State Examination. ^§^ NPI‐NH = Neuropsychiatric Inventory—Nursing Home version

This cross‐sectional study was performed using the baseline data from the *Co*mmunication, *S*ystematic pain treatment, *M*edication review, *O*rganized activities and *S*afety (COSMOS) study.^23^ Full details of the study protocol and procedures are described elsewhere.^23^ In brief, data were collected between August 2014 and December 2015. In total, 765 participants from 72 nursing home units from 37 nursing homes in Norway were included.^24^ The COSMOS study is a multicenter cluster randomized controlled trial that aims to improve quality of life in nursing home residents by enhanced communication (advanced care planning), proactive assessment and treatment of pain, discontinuation of unnecessary medication and organization of activities. Eligible criteria were all patients aged ≥65 years residing in long‐term care or a specialized dementia care unit without the diagnosis of schizophrenia and with a life expectancy ≥ 6 months, estimated by the treating physician. In this cross‐sectional study, only participants with cognitive impairment (Minimal Mental State Examination [MMSE] < 24), an available blood pressure measurement and a Neuropsychiatric Inventory—Nursing Home version (NPI‐NH) score (partially or complete) at baseline were included (Figure 1).

Verbal and written informed consent was obtained from the nursing home residents if they had sufficient ability to consent; if not, written presumed consent was obtained from a legally authorized representative, in accordance with the ethics committee requirements and current Norwegian legislation.

### 2.2 Measurements

Data collection was completed in close collaboration with a staff member who had been familiar with the participants for a minimum of 4 weeks prior to data collection. The staff received training in the appropriate use of each questionnaire and had assistance from the researchers as needed. Baseline data per participant on socio‐demographic characteristics (age, sex and civil status) were collected from the medical record. Cognitive performance was assessed at baseline using the MMSE.^25^ The classification of the MMSE was done on a 30‐point scale where from 0 to 11 points are classified as severe impairment, from 12 to 17 points as moderate impairment and from 18 to 23 points as mild cognitive impairment.^26^

### 2.3 Blood pressure

The baseline blood pressure measurement was done by the nurses in the units in mmHg according to the local procedure. SBP values were divided into two categories, low and high SBP. Since there is no evidence for the ideal blood pressure level in patients with dementia besides the ESH/ESC guidelines target SBP >130 mmHg, nor what the cut‐off point of a low SBP would be in those patients, we defined low SBP as a SBP <113 mmHg. This cut‐off was based on the first quartile of the SBP values at baseline.

### 2.4 Use of antihypertensive medication

Scheduled drug prescriptions (excluding prescriptions given “as needed”) were extracted from the participants' medical records. The antihypertensive drugs were assessed by counting the number of prescriptions for drugs classified as antihypertensive medication (Anatomical Therapeutic Chemical [ATC] code C03, C07‐C09).

### 2.5 Neuropsychiatric symptoms

NPS were assessed using the NPI‐NH, conducted by a trained research assistant based on a face‐to face interview with the caregiver who was familiar with the participant.^27^, ^28^ Screening questions are appraised about 12 different NPS, initially, and, when a symptom is present, followed by questions about the frequency and severity. The frequency is measured on a four‐point scale (higher score is more frequent; score of four points represents symptoms present on a daily basis) and severity on a three‐point scale (higher score is more severe; score of three points represents symptoms with much stress on the participant). For each of the 12 NPS, a total score was generated by multiplying the frequency and severity with a maximum of 12 resulting in a maximum total NPI‐NH score of 144 points. On a test–retest reliability study, correlation coefficients were 0.79 for overall frequency and 0.86 for overall severity. Studies have reported good inter‐rater reliability.^27^ The NPI‐NH has been cross‐validated with the NPI and is considered to be a brief, reliable, informant‐based assessment of NPS.^27^ The NPS were clustered according to the four‐factor model of Cheng et al.^29^ into the NPI clusters psychosis, mood, behavior problems and euphoria.

### 2.6 Statistical analysis

Baseline characteristics were described as mean with SD or median with interquartile ranges (IQR) for continuous variables and as numbers with percentage (%) for categorical variables for all participants. Baseline characteristics were also presented stratified for antihypertensive treatment use.

In our dataset, we had 25% missing values. To deal with missing values on the NPI‐NH (8.7%), socio‐demographic characteristics (5.5%) and pulse (1.0%), these were imputed using multiple imputations, creating 20 imputation sets.^30^ The posterior mean matching method was used. The following baseline variables were used to build the imputation model: age, sex, civil status, nursing home, systolic and diastolic blood pressure, pulse pressure, pulse, MMSE score, and the NPI frequency and severity score. The given total NPI‐NH scores and NPI clusters scores were not included as predictors in the imputation model to avoid instability of the model. Therefore, the total NPI score and the NPI cluster scores were recalculated after imputation. The NPI‐NH scores were log transformed due to strong skewing of the distribution.

First, the association between SBP (lowest quartile vs rest; predictor) and NPI‐NH score (total, clusters and single symptom score) as a measure of NPS (outcome) was assessed. Second, the association between use of antihypertensive medication (predictor) and NPI‐NH score (total, clusters and single symptom score) as a measure of NPS (outcome) was assessed. Linear mixed model analyses were performed on the imputed datasets which were summarized using Rubin's rule. To account for similarities within nursing home cluster, nursing home cluster was included as a random effect. The model was applied in the crude data (no adjustments) and further stepwise analyzed corrected for age, sex and MMSE score.

In sensitivity analysis, the association between continuous SBP values and NPI‐NH scores was assessed.

Analyses were completed using SPSS version 23 (IBM, Armond, NY, USA).

## 3 RESULTS

Of the 545 participants who were assessed for their eligibility to participate in the COSMOS study, we excluded 133 participants. Participants were excluded with a MMSE score > 23 (n = 27), missing data on the MMSE (n = 53) or SBP levels (n = 48), or the NPI‐NH was completely absent (n = 5) (Figure 1). As a result, 412 participants were included in this study.

Table 1 presents the characteristics of the participants. The mean age was 86.9 (SD 7.4) year and 304 participants (73.8%) were female. In total, 222 (53.9%) participants had a MMSE score < 11 points, indicating severe cognitive impairment. The mean SBP was 128 mmHg (SD 21.5), with a total NPI score of 10 (IQR 3‐23). Antihypertensive medication was used in 240 participants (53.3%).

**TABLE 1:** Baseline characteristics of participants overall and grouped by antihypertensive treatment

		Antihypertensive treatment	
	Total n = 412)	Yes (n = 240)	No (n = 172)
Females (n,%)	304 (73.8)	167 (69.6)	137 (79.7)
Age (SD)	86.9 (7.4)	87.5 (7.2)	85.9 (7.6)
Civil status (n,%)			
Married	91 (23.3)	50 (22.1)	41 (25.0)
Widow	251 (64.4)	153 (67.7)	98 (59.8)
MMSE (SD)			
Total score	10.2 (6.9)	11.4 (6.6)	8.6 (7.0)
Severe <11	222 (53.9)	116 (48.3)	106 (61.6)
Moderate 12‐17	116 (28.2)	74 (30.8)	42 (24.4)
Mild 18‐23	74 (18.0)	50 (20.8)	24 (14.0)
Blood pressure mmHg (SD)			
Systolic	128 (21.5)	127 (20.2)	129 (23.1)
Diastolic	71 (12.5)	71 (12.6)	72 (12.2)
Antihypertensive drugs (n,%)	240 (58.3)		
Diuretics		135 (32.8)	
β‐blocking		109 (26.5)	
Calcium channel blockers		40 (9.7)	
Renin angiotensin II blockers and ACE inhibitors		95 (23.1)	
NPI‐NH (IQR)			
Total score	10 (3–23)	8.5 (2‐20)	13 (4‐28)
Psychiatric cluster	0 (0–2)	0 (0–2)	0 (0‐3)
Mood cluster	4 (0‐12)	2 (0‐8)	3 (0‐12)
Behavior cluster	2 (0–9)	4 (0–10)	6 (1‐13)
Euphoria	0 (0–3)	0 (0–0)	0 (0‐0)

When comparing, the group using antihypertensive treatment were significantly older (*P* = 0.028), less females (*P* = 0.002) and had a significant higher MMSE score (*P* = 0.025) than the nonusers of antihypertensive treatment.

Table 2 shows that there was no association between total NPI‐NH score and low SBP compared to normal/high SBP (estimate 1.07, 95%CI 0.81‐1.41, *P* = 0.65). When adjusting for age, sex and MMSE score, the results were similar (estimate 1.07, 95%CI 0.82‐1.40, *P* = 0.62; Table 3).

**TABLE 2:** Crude difference in NPI score according to systolic blood pressure (>113 mmHg vs ≤113 mmHg)

				Antihypertensive treatment			
		Total (n = 412)		Yes (n = 240)		No (n = 172)	
NPI‐NH		Estimate^a^ (95%CI)	*P*‐values	Estimate^a^ (95%CI)	*P*‐values	Estimate^a^ (95%CI)	*P*‐values
Total NPI score		1.07 (0.81–1.41)	0.65	0.99 (0.68‐1.44)	0.97	1.27 (0.83‐1.95)	0.27
NPI psychosis		0.89 (0.72‐1.09)	0.27	0.88 (0.66‐1.15)	0.34	0.88 (0.63‐1.23)	0.46
NPI behavior		0.83 (0.64‐1.08)	0.16	0.43 (0.65‐1.29)	0.63	0.68 (0.44‐1.03)	0.07
NPI mood		0.99 (0.77‐1.27)	0.93	1.10 (0.79‐1.54)	0.56	0.83 (0.56‐1.23)	0.36
NPI euphoria		0.93 (0.85‐1.01)	0.09	0.96 (0.86‐1.06)	0.40	0.89 (0.76‐1.02)	0.13

Abbreviations: NPI‐NH = Neuropsychiatric Inventory—Nursing Home version.

^a^ Estimate calculated by mixed model analyses, transformed back after log transformation.

**TABLE 3:** Difference in NPI score according to systolic blood pressure (>113 mmHg vs ≤113 mmHg), adjusted for age, sex and MMSE score

				Antihypertensive treatment			
		Total (n = 412)		Yes (n = 240)		No (n = 172)	
NPI‐NH		Estimate^a^ (95%CI)	*P*‐values	Estimate^a^ (95%CI)	*P*‐values	Estimate^a^ (95%CI)	*P*‐values
Total NPI score		1.07 (0.82–1.40)	0.62	0.99 (0.68‐1.42)	0.95	1.32 (0.87‐1.99)	0.20
NPI psychosis		1.15 (0.93‐1.40)	0.19	1.13 (0.87‐1.48)	0.37	1.19 (0.86‐1.65)	0.29
NPI behavior		1.19 (0.92‐1.54)	0.18	1.07 (0.76‐1.49)	0.96	1.49 (0.97‐2.25)	0.05
NPI mood		1.01 (0.79‐1.30)	0.94	0.90 (0.64‐1.26)	0.55	1.20 (0.81‐1.77)	0.36
NPI euphoria		1.08 (1.03‐1.13)	0.08	1.04 (0.99‐1.09)	0.43	1.13 (0.97‐1.34)	0.12

Abbreviations: MMSE = Minimal Mental State Examination; NPI‐NH = Neuropsychiatric Inventory—Nursing Home version.

^a^ Estimate calculated by mixed model analyses, transformed back after log transformation.

There was no clinically significant association between low SBP and NPI‐NH clusters, in the crude data as well as in the adjusted model (Tables 2 and 3). Nor was there an association between low SBP and NPI clusters when stratified for antihypertensive medication use. Same analysis was done for all 12 single NPI items without a significant association found (data not shown).

The use of antihypertensive treatment was associated with a lower NPI‐NH score in the crude model (estimate 1.31, 95%CI 1.02‐1.68, *P* = 0.03, no use vs use; Table 4). On the contrary, in the adjusted model, the association between the use of antihypertensive treatment and the NPI‐NH score diminished (estimate 1.18; 95%CI 0.92‐1.51, *P* = 0.19; Table 5).

**TABLE 4:** Crude difference in NPI score according to the use of antihypertensive medication (no use vs use)

NPI‐NH		Estimate^a^ (95%CI)	*P*‐values
Total NPI score		1.31 (1.02‐1.68)	0.03
NPI psychosis		1.03 (0.86‐1.24)	0.73
NPI behavior		1.26 (1.00–1.59)	0.05
NPI mood		1.26 (1.01–1.58)	0.04
NPI euphoria		1.03 (0.96‐1.12)	0.40

Abbreviations: NPI‐NH = Neuropsychiatric Inventory—Nursing Home version.

^a^ Estimate calculated by mixed model analyses, transformed back after log transformation.

**TABLE 5:** Difference in NPI score according to use of antihypertensive medication (no use vs use) adjusted for age, sex and MMSE score

NPI‐NH		Estimate^a^ (95%CI)	*P*‐values
Total NPI score		1.18 (0.92–1.51)	0.19
NPI psychosis		1.01 (0.84‐1.22)	0.88
NPI behavior		1.15 (0.91‐1.46)	0.23
NPI mood		1.19 (1.06‐1.49)	0.14
NPI euphoria		1.01 (0.97‐1.06)	0.74

Abbreviations: MMSE = Minimal Mental State Examination; NPI‐NH = Neuropsychiatric Inventory—Nursing Home version.

^a^ Estimate calculated by mixed model analyses, transformed back after log transformation.

Separate analyses were done for the four NPI clusters. In the crude model, the NPI cluster behavior (estimate 1.26, 95%CI 1.00‐1.59, *P* = 0.05) and the mood cluster (estimate 1.26, 95%CI 1.01‐1.58, *P* = 0.04) both show a significant association with the no antihypertensive treatment group (Table 4). Conversely, no significant association was found between use of antihypertensive drugs and the NPI clusters in the adjusted analysis (Table 5).

In the sensitivity analyses with the continuouss SBP levels, there was no association between SBP and NPI‐NH score (estimate 1.00, 95%CI 0.98‐1.01, *P* = 0.25).

## 4 DISCUSSION

In this cross‐sectional study of nursing home residents with dementia, we did not find an association between low SBP and NPI‐NH total score, nor between the use or no use of antihypertensive medication and the NPI‐NH score.

Prior studies have identified cardiovascular disease as an important predictor of cognitive decline.^31^ Moreover, fluctuations in blood pressure have been recently identified as an important risk factor for cognitive decline. There are few studies assessing the association between blood pressure and NPS. Evidence has been found that NPS is related to hypoperfusion of the specific brain area's using functional brain imagining (FBI).^13^ Especially apathic syndromes and aggression are associated with hypoperfusion of specific brain areas assessed by single photon emission computed tomography (SPECT).^11^, ^14^, ^17^ The average MMSE score of the patient groups in these studies was much higher compared to our study (MMSE score from 17.6 to 22 vs 10.2) and the sample was smaller, 10 to 30 patients vs 412 patients. Blood pressure is not taken into account in these studies. Our study used low SBP as a hypothetic proxy variable (derivate) of hypoperfusion in the brain, which is less accurate than FBI or SPECT, but more clinically relevant in the nursing home setting.

A recent study found an association between the use of antihypertensive medication and a higher total NPI score.^22^ On the contrary, results from the crude model analysis of our study showed a significant lower NPI cluster behavior and mood score with antihypertensive treatment use. One possible explanation is the higher age and higher MMSE score in the group using antihypertensive medication. When adjusted for age, sex and MMSE score, there was no significant association between antihypertensive treatment and NPI‐NH score. In line with previous studies,^1^, ^32^, ^33^ the minimal clinical important difference of the NPI total score and the NPI single symptom score is greater than three points.^29^, ^34^ This cut‐off point was taken to indicate the presence of specific “clinically relevant” symptoms. We did not find a significant nor clinical relevant association between SBP or antihypertensive treatment and the NPI score.

In the group not using antihypertensive treatment, more NPS were found when SBP >113 mmHg (estimate 1.49, *P* = 0.05). This is contrary to our hypothesis and with the previous mentioned studies.^11^, ^14^

The mean SBP in our study was 128 mmHg and the 25th quartile (113 mmHg) was used as a cut‐off point. This mean SBP is assumed to be a low average for the frail elderly. In the literature, there is no clear or proven cut‐off point for low SBP in people of old age with comorbidities as dementia.^35^ Literature suggests a SBP below 120 mmHg in community‐dwelling people of old age is associated with an increased risk of cardiovascular events.^35^, ^36^, ^37^ It remains unclear if this applies to the nursing home population with dementia. Steinberg et al. assumed that a SBP below 128 mmHg and the use of antihypertensive treatment were associated with a greater decline in MMSE score and recommend a target SBP between 130 and 145 mmHg.^20^ The group that used antihypertensive treatment had on average a higher MMSE score than the group without antihypertensive treatment, on the contrary in our analyses. Since this is a cross‐sectional analysis, there was no information on cognitive decline.

A possible explanation is that physicians might be more prone to stop the antihypertensive treatment when the MMSE is low, as for other medications.^38^ Lower MMSE score, which is an indication of more cognitive decline and more severe dementia, is associated with NPS resulting in a higher NPI score. As a result, the use of antihypertensive drugs can be associated with less NPS, without being a causality. Another possible explanation is that using antihypertensive drugs might have a cardiovascular protective effect on the cognitive decline at old age.

A strength of the present study is our nursing home population, where studies in the frail and older population are scare. Research about blood pressure and/or the use of antihypertensive drugs in older people with dementia is scare and most of them are done in community‐dwelling elderly. Especially studies in nursing homes about NPS and blood pressure are lacking.

Furthermore, for measurement of NPS we used the NPI‐NH. It is considered to be a brief, reliable, informant‐based assessment of NPS.^27^

This study has some limitations. The SBP measurement was not standardized and extracted from the medical record.^23^ The blood pressure could have been measured in different ways (lying, sitting, by hand or automatic, on different times of the day). This was not reported nor formally protocolized. Also, this study is done in nursing home residence, so the results are not a good representation of the general population. Additionally, there can be a selection bias if a participant refused to cooperate regarding to his behavior. Furthermore, only the use (yes/no) of antihypertensive medication was known, not the dosage nor duration or indication. Therefore, dose—response could not be analyzed. An association between antihypertensive medication and NPI‐NH score could therefore potentially be missed. In addition, the use of psychotropic medication that could influence the occurrence of NPS could not be taken into account on our analysis.

It remains unclear if blood pressure is associated with NPS in persons with advanced dementia. The indication and duration of use of antihypertensive medication can play a role in the association with potential development of NPS, but even so cerebrovascular damage, blood pressure level or the use of (a type of) antihypertensive medication itself. Further prospective research is necessary to clarify the cardiovascular effects on the brain of persons with dementia and to have a good understanding about the etiology of NPS and treatment options, especially regarding blood pressure and the use of antihypertensive medication.

In conclusion, we found no evidence to confirm the hypothesis that low SBP and/or the use of antihypertensive medication are associated with more NPS in nursing home residents with dementia.

### Clinical Trail Registration

The COSMOS trial is approved by the Regional Committees for Medical and Health Research Ethics, 2013/1765, and registered at clinicaltrials.gov, NCT02238652.

## CONFLICT OF INTEREST

The authors have reported no conflicts of interest.

ACE = Angiotensin Converting Enzyme; MMSE = Minimal Mental State Examination; NPI‐NH = Neuropsychiatric Inventory—Nursing Home version.

## Data Availability

Data available on request due to privacy/ethical restrictions
